# Realization of Quasi‐Omnidirectional Solar Cells with Superior Electrical Performance by All‐Solution‐Processed Si Nanopyramids

**DOI:** 10.1002/advs.201700200

**Published:** 2017-07-06

**Authors:** Sihua Zhong, Wenjie Wang, Miao Tan, Yufeng Zhuang, Wenzhong Shen

**Affiliations:** ^1^ Institute of Solar Energy and Key Laboratory of Artificial Structures and Quantum Control (Ministry of Education) School of Physics and Astronomy Shanghai Jiao Tong University Shanghai 200240 P. R. China; ^2^ Collaborative Innovation Center of Advanced Microstructures Nanjing 210093 P. R. China

**Keywords:** high electric energy production, low carrier recombination, metal‐assisted alkaline etching, omnidirectional solar cells, Si nanopyramids

## Abstract

Large‐scale (156 mm × 156 mm) quasi‐omnidirectional solar cells are successfully realized and featured by keeping high cell performance over broad incident angles (θ), via employing Si nanopyramids (SiNPs) as surface texture. SiNPs are produced by the proposed metal‐assisted alkaline etching method, which is an all‐solution‐processed method and highly simple together with cost‐effective. Interestingly, compared to the conventional Si micropyramids (SiMPs)‐textured solar cells, the SiNPs‐textured solar cells possess lower carrier recombination and thus superior electrical performances, showing notable distinctions from other Si nanostructures‐textured solar cells. Furthermore, SiNPs‐textured solar cells have very little drop of quantum efficiency with increasing θ, demonstrating the quasi‐omnidirectional characteristic. As an overall result, both the SiNPs‐textured homojunction and heterojunction solar cells possess higher daily electric energy production with a maximum relative enhancement approaching 2.5%, when compared to their SiMPs‐textured counterparts. The quasi‐omnidirectional solar cell opens a new opportunity for photovoltaics to produce more electric energy with a low cost.

## 1 Introduction

When evaluating the performance of solar cells, it is more reasonable to examine their electricity generation ability in a day or a year rather than only in the standard test condition with normal incident light, because the incident angle (θ) changes as the sun goes from east to west in a day as well as moves between the Northern Hemisphere and the Southern Hemisphere in a year. Though photovoltaic sun tracking system is effective to solve the problem induced by the changed θ, it is costly and difficult to work precisely for more than 20–25 years of module lifetime.^1^ In recent years, there are lots of reports demonstrating that Si nanostructures possess outstanding broadband and omnidirectional antireflection ability, which are promising to be adopted in solar cells to form omnidirectional solar cells for enhancing electric energy output over broad θ.^2^, ^3^, ^4^, ^5^, ^6^, ^7^, ^8^, ^9^ For example, Spinelli et al.^2^ have reported that Si nanocylinder arrays can effectively suppress reflection over the wide θ of 0°–60°. Savin et al.^6^ even demonstrated that Si nanopillars textured solar cell brings 3% higher in daily energy production as compared to the conventional Si micropyramids (SiMPs)‐textured solar cell, which benefits from its better angular acceptance. Hence, considerable efforts have been focused on integrating Si nanostructures into various solar cells, such as diffused homojunction cells,^10^, ^11^, ^12^ heterojunction cells,^13^, ^14^ photo‐electrochemical cells,^15^ hybrid cells,^16^, ^17^, ^18^ carrier‐selective contact cells,^19^ and ultrathin crystalline Si (c‐Si) cells.^20^

Despite the optical advantage, the conversion efficiencies (ηs) of the most reported Si nanostructures‐textured solar cells were not improved as expected, the reason behind which is the degradation of electrical property incurred from the accelerated carrier recombination.^11^, ^21^ Because Si nanostructures such as nanopores, nanowires, nanoholes, nanowells, and nanopillars generally have large surface area enhancement and their surface is hard to be perfectly passivated by dielectric thin films, the photongenerated carriers are easily recombined through the dangling bonds on the unpassivated surface and thus surface recombination dramatically increases. Now, it is widely accepted that suppression of carrier recombination is one of the key points to improve the Si nanostructures‐textured solar cells.^21^

Recently, through passivating the surface by thermal SiO_2_, SiO_2_/SiN_x_:H dual layers, or atomic layer deposited Al_2_O_3_ layer, ηs approaching or higher than 20% have been demonstrated on Si nanostructures‐textured solar cells thanks to the excellent passivation effect of these dielectric layers.^6^, ^22^, ^23^ Nevertheless, when compared to their conventional SiMPs‐textured counterparts, the nanostructures‐textured solar cells still suffer from higher carrier recombination and thus have lower open‐circuit voltage (*V*_OC_). In fact, besides utilizing outstanding passivation layers, developing Si nanostructures with low surface enhancement is also an effective approach to reduce carrier recombination.^21^ On this aspect, Si nanopyramids (SiNPs) have extremely attracted interests due to the fact that they have low surface enhancement, while excellent antireflection and light trapping effect are retained.^24^, ^25^, ^26^, ^27^ Therefore, SiNPs have been successfully demonstrated to bring benefits for solar cells, especially in ultrathin c‐Si solar cells.^28^, ^29^, ^30^ However, we also notice that the knowledge on the angle‐dependent optical performance of SiNPs is very limited and it is also unknown whether the design of SiNPs is superior to SiMPs on solar cells.

In this study, SiNPs were employed as surface texture due to their potential of lower carrier recombination as compared to other Si nanostructures. We fabricated SiNPs on large‐scale (156 mm × 156 mm) wafers via our recently proposed metal‐assisted alkaline etching (MAAE) method,^26^ which is an all‐solution‐processed method and does not involve patterning process as required in the present popular methods.^27^, ^30^, ^31^, ^32^ We have excitingly found that the SiNPs can be easily passivated by intrinsic hydrogenated amorphous Si (i a‐Si:H) layer or SiN_x_:H layer and exhibit lower surface recombination compared to the conventional SiMPs counterparts, which is attributed to the lower surface enhancement. As a result, superior electrical properties are achieved in both SiNPs‐textured homojunction and heterojunction solar cells. Moreover, it is found that SiNPs‐textured solar cells are actually quasi‐omnidirectional solar cells and thus have very little drop of quantum efficiency (QE) with increasing θ, which benefits from their excellently broad‐angle antireflection property over the θ of 0°–50°. Via the careful calculation with considering the variation of solar spectrum with time, relative enhancement of daily output energy between 1.2% to nearly 2.5% is expected depending on the seasons when compared to conventional SiMPs‐textured counterparts.

## 2 Results and Discussion

### 2.1 Formation of SiNPs

**Figure 1:**
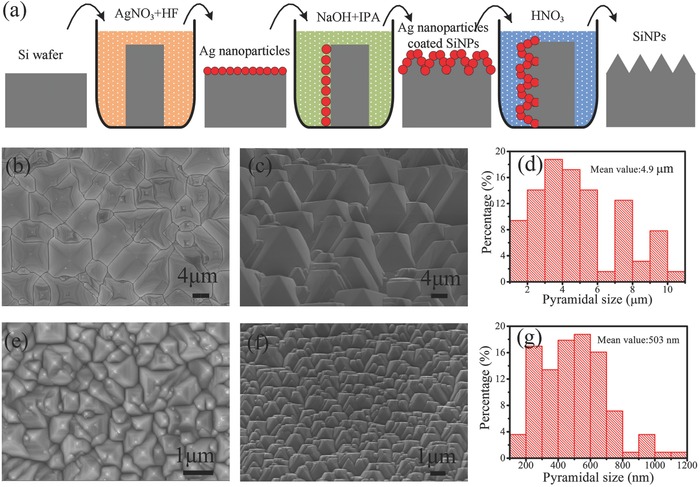
a) Schematic diagram of the fabrication process of SiNPs by MAAE method. b) Top‐view, c) 45° tilted‐view SEM images and d) lateral size distribution of the conventional SiMPs texture. e) Top‐view, f) 45° tilted‐view SEM images, and g) lateral size distribution of the SiNPs texture.

**Figure** **1**a schematically shows the fabrication process of SiNPs, namely the deposition of Ag nanoparticles in AgNO_3_/HF solution at first, then etching in alkaline solution under 60 °C for 20 min, followed by the removal of Ag nanoparticles in HNO_3_ solution. More details of the fabrication are presented in the Experimental Section. Compared to the present popular methods to form SiNPs, the MAAE method does not require etching pattern and thus does not involve lithography process or ion etching.^27^, ^30^, ^31^, ^32^ It only requires the deposition of Ag nanoparticles in solution before alkaline etching and is an all‐solution‐processed method. Evidently, it is highly simple and cost‐effective. Moreover, the MAAE method can be easily applied to large‐sized Si wafers, such as 156 mm × 156 mm, and produces uniform surface texture, as presented in Figure S1 (Supporting Information). Therefore, the MAAE method is compatible with the mass production lines of c‐Si solar cells. As the surface reference texture, SiMPs were formed by the conventional texturing method, which is described in the Experimental Section and can be easily found in literatures.^33^, ^34^

The typical top‐view and 45° tilted‐view scanning electron microscopy (SEM) images together with the pyramidal size distribution of the SiMPs and SiNPs are shown in Figure 1b–g. One can observe that both the SiMPs and the SiNPs are densely distributed no matter from top view or 45° tilted view, exhibiting perfect surface textures. The sizes of the SiMPs range from several hundred nanometers to over 10 µm with a mean value of 4.9 µm. The sizes of SiNPs are distributed from 100 to 1200 nm but concentrated in 200–800 nm with a mean value of 503 nm. It is worth mentioning that the sizes of the SiNPs are not sensitive to the etching temperature.^26^ Besides the size difference, the SiNPs have much smoother surface, which is beneficial for reducing surface recombination as will be presented in next part.

**Figure 2:**
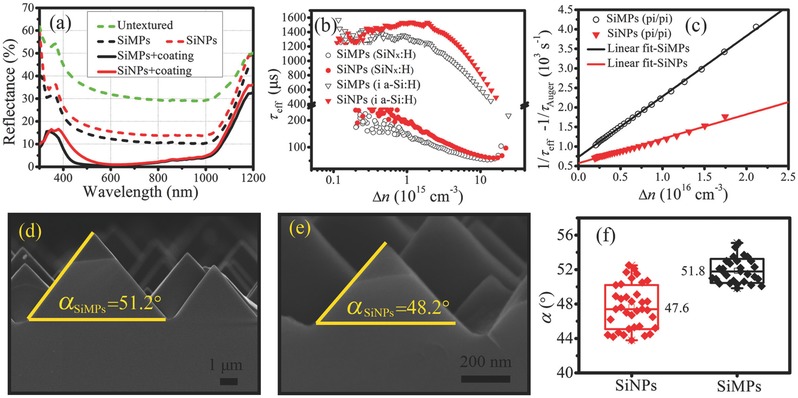
a) Reflectance spectra of the untextured, SiMPs‐textured, and SiNPs‐textured surfaces with and without antireflection coating. b) Comparison of τ_eff_ of SiMPs‐textured and SiNPs‐textured samples, which are symmetrically passivated by either i a‐Si:H film or SiN*_x_*:H film. c) Comparison of (1/**τ**_eff_ − 1/**τ**_Auger_) of the SiMPs‐textured and the SiNPs‐textured samples symmetrically coated by p/i a‐Si:H films. Also shown is the linear fits of (1/**τ**_eff_ − 1/**τ**_Auger_) versus Δ*n*, through which *J*_0e_s are determined. d) Cross‐sectional SEM image of SiMPs. e) Cross‐sectional SEM image of SiNPs. f) Comparison of the distribution of αs of the SiMPs and SiNPs.

**Figure** **2**a demonstrates that both the SiMPs texture and SiNPs texture effectively reduce the reflectance as compared to the untextured surface over the whole wavelength range, but due to different antireflection mechanisms. The SiMPs reduce reflectance through the multiple reflections of incident light and thus enhancing the chance of refracting into the Si bulk. For the SiNPs, because their sizes are comparable to the light wavelength, which is beyond the geometric optics theory, they inhibit surface reflectance by efficiently coupling the incident light into the substrate via optical resonance and light scattering. For the uncoated samples, the reflectance of the SiNPs‐textured wafer is higher than that of the SiMPs‐textured one over the whole wavelength range. However, after the deposition of In_2_O_3_:W as antireflection layer, reflectance of the both samples greatly declines and the gap between them greatly shrinks. This phenomenon is also observed in SiN_x_ layers coated samples.^26^ The solar spectrum averaged reflectance of the coated SiNPs is 3.6% over the wavelength of 400–1100 nm, which is slightly higher than that of the coated SiMPs (2.5%).

### 2.2 Superior Suppression of Recombination

Figure 2b compares the effective minority carrier lifetime (τ_eff_) of the SiMPs‐textured and SiNPs‐textured n‐type c‐Si samples as a function of injected carrier density (Δ*n*). The SiNPs‐textured wafers possess higher τ_eff_ than the SiMPs‐textured wafers no matter using i a‐Si:H or SiN_x_:H as passivation layer. For instance, at Δ*n* of 1 × 10^15^ cm^−3^, *τ_eff_s for the SiN_x_*:H film passivated SiMPs and SiNPs are 145 and 194 µs, respectively, and τ_eff_s are 1314 and 1521 µs for the i a‐Si:H passivated SiMPs‐textured and SiNPs‐textured samples, respectively. According to the τ_eff_, surface recombination velocity (*S*_eff_) can be determined by the following equation^35^

(1) $$\frac{1}{\tau_{\mathrm{eff}}}=\frac{1}{\tau_{\mathrm{bulk}}}+\frac{2S_{\mathrm{eff}}}{W}$$

where *W* is the wafer thickness (180 µm), τ_bulk_ the bulk recombination lifetime. Here, we only consider the intrinsic τ_bulk_ according to the formula by Richter et al.^36^ and thus the calculated *S_eff_ represents the upper limit of the surface recombination velocity. For the SiN_x_*:H film passivated SiMPs and SiNPs, *S*_eff_s are 61.8 and 46.2 cm s^−1^, respectively, and for the i a‐Si:H passivated SiMPs and SiNPs, *S*_eff_s are 6.6 and 5.7 cm s^−1^, respectively. Both the results demonstrate that the SiNPs‐textured surfaces suffer lower surface recombination.

To evaluate the carrier recombination on the different surface textures after the formation of emitter, we have also measured the τ_eff_ of the n‐type c‐Si wafers with p/i a‐Si:H layers symmetrically deposited on both sides to extract emitter saturation current density (*J*_0e_) based on the following equation^37^

(2) $$\frac{1}{\tau_{\mathrm{eff}}}-\frac{1}{\tau_{\mathrm{Auger}}}=\frac{1}{\tau_{\mathrm{SRH}}}+\frac{2J_{0e}}{qn_{i}^{2}W}(N_{\mathrm{dop}}+\Delta n)$$

where τ_Auger_ and τ_SRH_, respectively, represent the carrier lifetime related to Auger recombination and Shockley–Read–Hall recombination, *q* is the electron charge, *n*_i_ is the intrinsic carrier density, and *N*_dop_ is the doping concentration of the substrate. Through the linear fit of (1/**τ**_eff_ − 1/**τ**_Auger_) versus Δ*n*, as shown in Figure 2c, *J*_0e_ is calculated to be 9 fA cm^−2^ for the SiNPs‐textured sample, much lower than that of the SiMPs‐textured sample (22 fA cm^−2^). As it is known, *J*_0e_ indicates the carrier recombination in the emitter and it is inversely proportional to the *V*_OC_ of solar cells. The lower *J*_0e_ of the SiNPs‐textured sample indicates lower carrier recombination in the emitter and will yield a higher *V*_OC_.

It is intriguing that both the microtexture and nanotexture are pyramid‐like morphology but the τ_eff_ is higher and the *J*_0e_ is lower for the nanotextured samples. In order to get insight into the difference on carrier recombination, we have further studied cross‐sectional SEM images of the SiMPs and SiNPs. As presented in Figure 2d,e, the base angles (αs) of the SiMPs and the SiNPs are 51.2° and 48.2°, respectively, exhibiting lower α for the SiNPs. This is not an individual case. Through measuring lots of α, it is found that the averaged α of the SiNPs is 47.6°, which is indeed lower than that of the SiMPs (51.8°), as shown in Figure 2f. Here, it is worth mentioning that the α of our SiMPs lower than 54.7° (the α of a standard pyramid) is coincident with the previously reported results (the typical industrial SiMPs have αs between 49° and 53°).^38^, ^39^ The investigation of the exact reasons for the different αs between the SiMPs and the SiNPs is outside the scope of this work, but one of the possible reasons is that the SiNPs are fabricated in a lower solution temperature (the temperatures are 60 and 83 °C for fabricating the SiNPs and the SiMPs, respectively), which reduces the difference of etching rates between 〈100〉 and 〈111〉 crystallographic orientation.^40^ As exhibited in Figure S2 (Supporting Information), higher ecthing temperature can lead to higher αs. Based on 3D geometry and the α, the surface enhancement ratio of the SiMPs is calculated to be 1/(cos51.8°) = 1.62, and that of the SiNPs is 1/(cos47.6°) = 1.48. Therefore, the SiNPs have lower surface area. We have confirmed this conclusion by measuring the mass and the thickness of the deposited SiN_x_ layer to calculate the textured surface area and found that the surface area of the SiNPs‐textured wafer is only 0.86 times that of the SiMPs‐textured wafer. It should be noted that the smoother surface of the SiNPs is also beneficial to reduce the surface area, which explains why the measured ratio (0.86) is lower than the calculated one (1.48/1.62 = 0.91). Since the passivation layer is impossible to fully passivate the surface dangling bonds, hence lower surface area indicates lower surface dangling bonds and thus lower surface recombination, which is responsible for the higher τ_eff_ and lower *J*_0e_.

### 2.3 Solar Cell Performances

**Figure 3:**
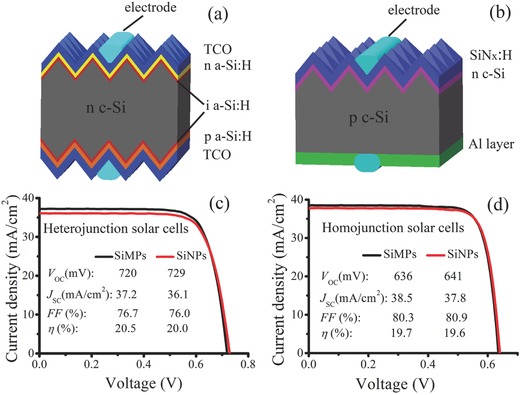
Schematic diagrams of a) heterojunction solar cell and b) homojunction solar cell. Comparisons of *J*–*V* characteristics of the SiMPs‐textured and the SiNPs‐textured solar cells with c) heterojunction structure and d) homojunction structure.

We have fabricated both homojunction and heterojunction solar cells on large‐sized (156 mm × 156 mm) wafers to evaluate the influence of surface textures on the performances of solar cells. For heterojunction solar cells, as shown in **Figure** **3**a, the used substrates are n‐type c‐Si wafers. P a‐Si:H/i a‐Si:H and n a‐Si:H/i a‐Si:H stack layers are deposited on the both sides of the substrates, respectively, and then In_2_O_3_:W is deposited on the both sides as transparent conductive oxide (TCO) layers and antireflection layers. For homojunction solar cells (Figure 3b), the used substrates are p‐type c‐Si wafers and the solar cell structure is the most common one in industry without special advanced techniques. Namely, the front side (illumination side) is doped by the diffusion of phosphorus to form n‐type emitter and then covered by SiN_x_:H layer acting as both passivation layer and antireflection layer. The back side is directly covered with Al layer to provide back surface field.

Figure 3c,d compares the current density–voltage (*J*–*V*) characteristics of the typical SiMPs‐textured and the SiNPs‐textured solar cells (their averaged performances are listed in Table S1 in the Supporting Information). As expected, the *V*_OC_ of the SiNPs‐textured solar cells is higher than that of the SiMPs‐textured counterparts in no matter homojunction structure or heterojunction structure, which benefits from the lower surface carrier recombination of the SiNPs. Moreover, due to the excellent passivation quality of the a‐Si:H stack layers, the *V*_OC_ of the SiNPs‐textured heterojunction solar cells even reaches as high as 729 mV, which is the highest *V*_OC_ of nanostructured solar cells as far as we know. With respect to fill factor (*FF*), the SiNPs texture also shows an (potential) advantage. It is reported that reducing the surface recombination to increase carrier lifetime is beneficial for improving *FF*.^41^, ^42^ Indeed, the *FF* of the SiNPs‐textured homojunction solar cell is 0.6% absolutely higher than that of the SiMPs‐textured one. For heterojunction solar cells, although the actual *FF* obtained from the *J*–*V* curve is lower for the SiNPs‐textured one than its counterpart, the pseudo *FF* measured from Suns‐*V*_OC_ is higher (pseudo *FF* of the SiMPs‐textured and SiNPs‐textured heterojunction cells are 79.7% and 81.7%, respectively, which are shown in Figure S3 in the Supporting Information). Note that the Suns‐*V*_OC_ measurement excludes the influence of series resistance, and hence it intrinsically reflects the limit of *FF*. Therefore, the lower actual *FF* of the SiNPs‐textured heterojunction solar cell is ascribed to the higher series resistance loss that may come from the thicker i a‐Si:H layers on the both sides because lower surface area generally leads to thicker deposition layer in plasma enhanced chemical vapor deposition (PECVD) system. An example that can be easily observed is the deposition of SiN_x_:H film (see Figure S4 in the Supporting Information). Since this is not the intrinsic problem of the SiNPs, its actual *FF* is expected to be higher by further optimizing the thickness of i a‐Si:H layer. Owing to the slightly higher surface reflectance, both the SiNPs‐textured homojunction and heterojunction solar cells have lower short‐circuit current density (*J*_SC_) than their respective counterparts. The difference of *J*_SC_ in heterojunction solar cells is more obvious because the SiNPs‐textured cell suffers severer parasitic absorption loss due to the thicker a‐Si:H layers. As the overall results, the η of the SiNPs‐textured homojunction solar cell is comparable to that of the SiMPs‐textured one, while the η of the SiNPs‐textured heterojunction solar cell is a little lower than its counterpart.

It is well known that Si nanostructures‐textured solar cells generally suffer higher electrical loss than their SiMPs‐textured counterparts, which originates from their dramatically enhanced carrier recombination. However, our result demonstrates that Si nanostructures can also be used to realize superior electrical performances than the SiMPs, the key point for which is the fabrication of the Si nanostructures with lower surface enhancement. It is worth mentioning that for two kinds of solar cells with a comparable η, higher *V*_OC_ is more welcome than higher *J*_SC_ because it incurs lower energy loss in modules. Moreover, in the stage of proof‐of‐concept, the SiNPs‐textured solar cells were not sufficiently optimized yet. Their performances are believed to be further improved by optimizing the parameters of each layers, such as the thickness of a‐Si:H layers and TCO layers for heterojunction solar cells as well as the thickness of SiN_x_:H layers for homojunction solar cells.

### 2.4 Quasi‐Omnidirectional Solar Cell Characteristics

**Figure 4:**
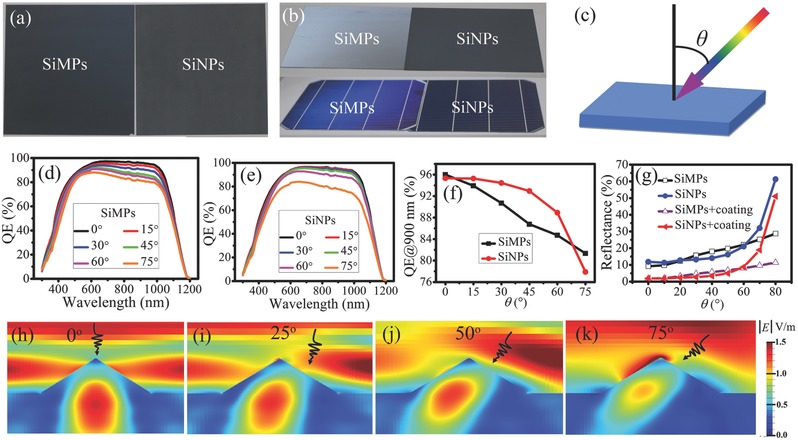
Digital photographs of SiMPs‐textured and SiNPs‐textured wafers taken under a) normal angle and b) tilted angle. Also shown is the photograph of the finished solar cells under tilted angle. c) Definition of the incident angle θ in the study. QE spectra of d) SiMPs‐textured and e) SiNPs‐textured heterojunction solar cells varying with θ together with f) comparison of their QE values at 900 nm. g) Simulated reflectance of SiMPs and SiNPs versus θ with and without antireflection coating. h–k) Light intensity within single SiNP and substrate with different θ. *E* represents electric field intensity. Incident directions of light are schematically denoted by the arrows.

As discussed above, the lower *J*_SC_s of the SiNPs‐textured solar cells are attributed to their slightly higher reflection loss. However, it should be noted that these performances were measured under normal incident light. Interestingly, when we look at the Si wafers, the SiMPs‐textured one exhibits black from top view and it becomes a little shining from tilted angle view, whereas the SiNPs‐textured one looks black no matter from top view or tilted angle view, as shown in **Figure** **4**a,b. Moreover, these characteristics still exist even for the finished solar cells, showing the potential of SiNPs to form omnidirectional solar cells that are characterized by excellently broad‐angle acceptance on photoelectric conversion.

To confirm this, we have measured the QEs of both the SiMPs‐textured and SiNPs‐textured solar cells (including both the homojunction and heterojunction structure) by varying the θ. Figure 4c specifies the definition of θ in the study, which is equal to the intersection angle between the incident light direction and the normal direction of solar cell. For heterojunction solar cells, from Figure 4d, it can be seen that the QE spectra of the SiMPs‐textured solar cell decrease gradually with increasing θ. Differently, Figure 4e shows that when the θ is smaller than 45°, the QE spectra of the SiNPs‐textured solar cell drop very slightly with increasing θ, but when the θ is larger than 60°, it turns to drop rapidly with increasing θ, which is similar to that of Si nanopillars‐textured solar cell.^6^ For better comparison, we have also plotted the QEs at 900 nm of the both kinds of solar cells as a function of θ, as shown in Figure 4f. It can be seen that the QE of the SiMPs‐textured solar cell decreases almost linearly with increasing θ. Nevertheless, the QE of the SiNPs‐textured one decreases much slowly with increasing θ in the region of 0°–45°, but outside the region, its decrease accelerates and becomes sharply when θ is larger than 60°. As a result, although the QE of the SiNPs‐textured solar cell is lower than that of the SiMPs‐textured one at the θ of 0°, it becomes higher at the 15° ≤ θ ≤ 60°. Figure S5 (Supporting Information) further demonstrates that the *J*_SC_s of the SiNPs‐textured cell calculated from the QE curves also change more slowly over broad θs. Moreover, it should be noted that these QE behaviors versus θ are the same for the homojunction solar cells, as presented in Figure S6 (Supporting Information). Such excellent spectrum response over broad θs indicates that the SiNPs‐textured solar cells are indeed quasi‐omnidirectional solar cells.

We have further carried out simulation of reflectance varying with θ to gain insight into the mechanism behind the quasi‐omnidirectional solar cells. As presented in Figure 4g, the reflectance of the SiMPs‐textured wafer at the wavelength of 900 nm increases linearly with θ, which is similar to the reported results,^5^, ^43^ while that of the SiNPs‐textured wafer increases very slightly with increasing θ in the range of 0°–50° and turns to increase dramatically when the θ is larger than 60°. After antireflection coating, although the reflectance of both the SiMPs‐textured and the SiNPs‐textured wafers are greatly reduced, their reflectance behaviors varying with θ are the same with the uncoated ones. It is obviously found that the reflectance and QE behaviors varying with θ are nearly coincident. Therefore, for the SiNPs‐textured solar cells, their quasi‐omnidirectional property on QE originates from their excellent antireflection over broad θs. To the best of our knowledge, this is the first study demonstrating that SiNPs can effectively reduce the surface reflectance over broad θs. This advantage is attributed to that their pyramidal sizes are comparable to the wavelength of incident light. We have theoretically investigated the interaction of incident light with single SiNP by varying the θ. Figure 4h–k indicates that incident light is efficiently coupled into the substrate by optical resonance and light scattering provided by the SiNP, which is beneficial for reducing the surface reflectance. Moreover, this optical resonance attenuates very slightly with increasing θ in the range of 0°–50°, but when the θ increases to 75°, dramatic attenuation of optical resonance is observed. These explain the reflectance behaviors varying with θ very well.

### 2.5 Higher Electric Energy Production

**Figure 5:**
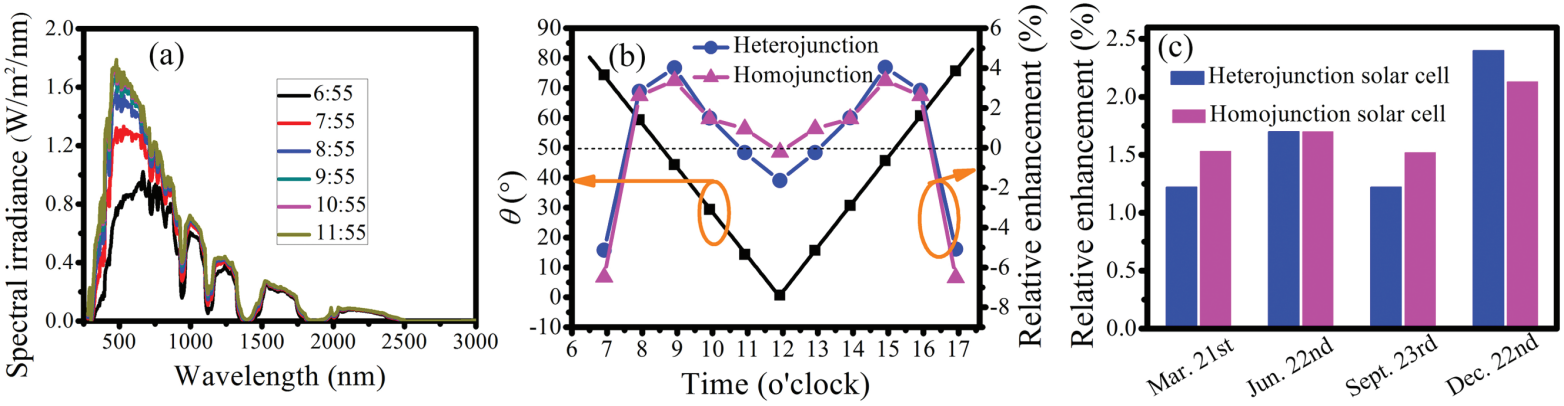
a) Solar spectral irradiance varying with time. b) θ and relative enhancement of the *P*_out_ of SiNPs‐textured solar cells to that of SiMPs‐textured solar cells as a function of time on September 23rd. Both the homojunction and heterojunction structures are included. c) Relative enhancement of energy output by both SiNPs‐textured homojunction and heterojunction solar cells on March 21st, June 22nd, September 23rd, and December 22nd, using SiMPs‐textured solar cells as references.

The characteristic of quasi‐omnidirectional solar cells is of paramount importance, since in real application, the sun goes from east to west, which results in the change of θ. To evaluate this merit, we have compared the output power (*P*_out_) of the SiMPs‐textured and SiNPs‐textured both homojunction and heterojunction solar cells over a day and a year by calculations with taking Shanghai (latitude of 31°) as an example of module location. The solar spectral irradiance and θ varying with time were extracted from solar spectrum calculator. The modules were fixed at latitude tilt and faced due south. As an example, **Figure** **5**a,b shows the solar spectral irradiance and θ varying with time on September 23rd, respectively. Based on these two parameters and the measured QEs together with neglecting the variation of temperature, *J*_SC_ varying with θ can be calculated by

(3) $$J_{\mathrm{SC}}(\theta )=q\int \mathrm{QE}(\lambda ,\theta )\Gamma (\lambda ,\theta )\cos\theta d\lambda$$

with Γ the incident photon flux calculated from the solar spectral irradiance and λ the wavelength. *V*_OC_ varying with θ is estimated by

(4) $$V_{\mathrm{OC}}(\theta )\approx \frac{kT}{q}\ln\left(\frac{J_{\mathrm{SC}}(\theta )}{J_{0}}\right)$$

where *k* is the Boltzmann constant, *T* is the temperature in Kelvins and is assumed to be constant at room temperature, *J*_0_ is the saturated current density and can be obtained under the case of θ = 0°. Given that *FF* is constant, *P*_out_(θ) is calculated by

(5) $$P_{\mathrm{out}}(\theta )\;=\;V_{\mathrm{OC}}(\theta )J_{\mathrm{SC}}(\theta )FF$$

Therefore, compared to the SiMPs‐textured solar cells, the relative enhancement of *P*_out_ by SiNPs‐textured solar cells varying with time or θ can be obtained and presented in Figure 5b. Clearly, although the *P*_out_ of the both SiNPs‐textured homojunction and heterojunction solar cells are lower than their corresponding SiMPs‐textured counterparts at θ of 0°, the *P*_out_ of the SiNPs‐textured solar cells become higher over the θ of 15°–60° and 30°–60° for homojunction and heterojunction, respectively, owing to their quasi‐omnidirectional advantage. As the consequence, both the SiNPs‐textured homojunction and heterojunction solar cells have higher *P*_out_ most of the time and thus generate more electric energy over a day. Figure 5c shows the relative enhancement of the electric energy production on March 21st, June 22nd, September 23rd, and December 22nd, which are the representative days of the spring, summer, autumn, and winter seasons of the Northern Hemisphere. From this figure, it can be expected that both the SiNPs‐textured homojunction and heterojunction solar cells generate more electric energy in any season and especially in winter season, the relative enhancement is the highest, approaching 2.5%. We believe that the relative enhancement can be higher if the quasi‐omnidirectional solar cells are further optimized.

## 3 Conclusions

We have successfully realized large‐sized (156 mm × 156 mm) quasi‐omnidirectional solar cells with SiNPs as surface textures. The SiNPs were fabricated by an MAAE method without involving any lithography and ion etching process to make patterns, which is an all‐solution‐processed method and highly simple together with cost‐effective. The SiNPs‐textured wafers exhibit higher τ_eff_ and lower *S_eff_ compared to the SiMPs‐textured counterparts no matter using i a‐Si:H films or SiN_x_*:H films as passivation layers. Moreover, after the formation of pn junction, its *J*_0e_ is also lower. These benefit from the lower surface enhancement of the SiNPs. As the result of lower carrier recombination, we have successfully realized higher *V*_OC_ and (peudo) FF for both homojunction and heterojunction solar cells, explicitly demonstrating that superior electrical performances can also be achieved in Si nanostructures‐textured solar cells. Furthermore, the SiNPs‐textured solar cells have very little drop of QE with increasing θ due to their excellent antireflection ability over θ of 0°–50°, which demonstrates the realization of quasi‐omnidirectional solar cells. Eventually, both SiNPs‐textured homojunction and heterojunction solar cells have higher daily output energy than the SiMPs‐textured counterparts. We believe that the SiNPs texture fabricated by the all‐solution‐processed method is promising to be adopted in photovoltaic industry to realize quasi‐omnidirectional solar cells for boosting electric energy production with a low cost.

## 4 Experimental Section

*Surface Textures*: The SiNPs textures were fabricated by an MAAE method.^26^ Figure 1a schematically shows the fabrication process. First, Ag nanoparticles were deposited on Si wafers by immersing them in a mixed solution of 5 × 10^−3^ m AgNO_3_ and 9 vol% HF for 5 s. Then the Si wafers were etched in an alkaline solution containing 1.1% NaOH and 8 vol% IPA under the solution temperature of 60 °C for 20 min, forming SiNPs coated with Ag nanoparticles. In the following, these Ag nanoparticles were removed by immersing in HNO_3_ solution to obtain the pure SiNPs. It should be noted that Si wafers were rinsed in deionized water between every two steps to remove the residual solution. Clearly, there are only three steps to form the SiNPs and it is very simple. For the fabrication of SiMPs texture, the conventional method was followed, namely, Si wafers were etched in an alkaline solution containing 1.1% NaOH and 8 vol% IPA under the solution temperature of 83 °C for 30 min.

*Fabrication of Solar Cells*: For heterojunction solar cells, n‐type Czochralski (CZ) Si wafers with sizes of pseudo‐square 156 mm × 156 mm and a resistivity of about 3.4 Ω cm were used as substrates. These Si wafers were polished in NaOH solution, and textured according to the above texturing methods to respectively form SiNPs and SiMPs (final wafer thickness is about 180 µm), and wet‐chemically cleaned to remove metal ion contamination, and dipped in HF solution to remove oxide layer, successively. Then, (≈10 nm) p a‐Si:H/(≈7 nm) i a‐Si:H and (≈10 nm) n a‐Si:H/(≈7 nm) i a‐Si:H stack layers were deposited on the back side and the front side of the wafers, respectively, in PECVD system. In the following, In_2_O_3_:W films with thicknesses of 80 nm were deposited onto the both sides as TCO layers by reactive plasma deposition system. Finally, electrodes were screen printed on the both sides with much dense grid lines on the back side (hence illumination from back side can be neglected) by a low‐temperature Ag paste, and then were sintered at 200 °C. Schematic diagram of the heterojunction solar cell is shown in Figure 3a.

For homojunction solar cells, p‐type CZ Si wafers with sizes of pseudo‐square 156 mm × 156 mm and a resistivity of about 1–3 Ω cm were used as substrates. These Si wafers were polished in NaOH solution, and textured according to the above texturing methods to respectively form SiNPs and SiMPs (final wafer thickness is about 180 µm), and wet‐chemically cleaned, and dipped in HF solution, successively. Then, these cleaned wafers were put into a diffusion tube furnace to receive n‐type diffusion using POCl_3_ as the dopant source, resulting in the sheet resistance of 95 Ω sq^−1^. In the following, the phosphorosilicate glass on the back side was etched by HF solution on single‐side cleaning equipment, and then the wafers were immersed in NaOH solution to polish the back side, followed by the removal of the phosphorosilicate glass on the front side in HF solution. Subsequently, SiN_x_:H layers with a thickness of 80 nm were deposited onto the wafer surfaces as passivation and antireflection coatings by PECVD. At last, the Al back surface field formation together with the metallization of the front and back electrodes were carried out with the screen printing technique, and then cofired in a conveyer belt furnace. Schematic diagram of the homojunction solar cell is shown in Figure 3b.

*Fabrication of Samples for Recombination Comparison*: For *τ_eff_ study, both sides of the SiMPs‐textured and SiNPs‐textured n‐type samples were symmetrically passivated by either (≈50 nm) i a‐Si:H films or (≈80 nm) SiN_x_*:H layers. For the extraction of *J*_0e_, both sides of Si wafers were symmetrically deposited with (≈10 nm) p a‐Si:H/(≈7 nm) i a‐Si:H stack layers. Note that, annealing at a temperature of 225 °C is required to improve the passivation quality of a‐Si:H films.

*Characterization*: The microstructures of the surface textures were investigated by field emission scanning electron microscopy. The pyramidal size distributions were obtained in Nano Measurer software by measuring the distance between two opposite base edges. The photographs of the Si wafers and solar cells were taken by a digital camera under fluorescent lamp. The reflectance of the as‐textured and TCO coated samples were measured with QEX10 (PV Measurements) system using integrating sphere attachment. Quantum efficiencies varying with θ were also measured in the QEX10 system with the measurement mode of quantum efficiency. Specially, a multifunction gradiometer was attached on the sample platform through magnetic attachment to monitor the θ. Minority carrier lifetime measurements were carried out with WCT‐120 (Sinton) equipment using quasi‐steady state photoconductance decay mode. The current–voltage tester was used to characterize the performances of the finished solar cells under AM1.5 spectrum and a temperature of 25 °C. Suns‐*V_OC_ equipment (Sinton) was used to obtain the pseudo FF of the heterojunction solar cells. In experiment, the surface ratio of SiNPs to SiMPs was determined by carefully measuring the mass of SiN_x_*:H layer before and after the removal of SiN_x_:H with HF solution and measuring the thickness of the SiN_x_:H layer through SEM.

*Simulations*: The surface reflectance together with electrical field intensity varying with θ was numerically calculated by Lumerical finite difference time domain (FDTD) software. 51 SiNPs were randomly distributed on a position of 3000 nm × 3000 nm square region on *x*–*y* plane with pyramidal sizes of 200–900 nm. Note that some pyramids were partly overlapped as the experimental ones and Bloch boundary in the *x*–*y* region was adopted because non‐normal incident case was included (θ was changed from 0°–80°). The ratio of the height to the half bottom length of the Si pyramids was fixed to be 1.11, corresponding to the α of 48°. The substrate thickness was set to be 1000 nm, but the boundary in the *z*‐directions was perfectly matched layers and hence the thickness of the substrate was equivalent to infinitely thick. For the case with antireflection layer, SiN_x_ film with a constant refractive index of 2 and a thickness of 75 nm was covered on the surface of SiNPs. Optical constant of c‐Si was directly extracted from the simulation software. The light source was a plane wave with a fixed wavelength of 900 nm (in the case of non‐normal incidence, the wavelength has to be fixed) and its polarization angle was set to be 45° as the consequence of averaging P polarization and S polarization. For studying the interaction between light and SiNP, single Si nanopyramid with a lateral size of 500 nm on a substrate was modeled with changing θ from 0°–75° to extract the light intensity within Si as a function of θ.

Owing to the significantly increased simulation time and the highly requirement for the computer for FDTD modeling SiMPs, the reflectance of the SiMPs texture was calculated by OPAL2, which is a professional optical simulator for the Si microstructures‐textured solar cells.^43^ The surface morphology was set to be random upright pyramids with α of 52°. Substrate thickness was 500 µm. Light trapping model was the default one. Refractive index of SiN_x_ film was also set to be a constant value of 2 without considering extinction coefficient. θ was varied from 0°–80°.

*Simulation of Solar Spectrum*: The solar spectrum irradiances under clear‐sky conditions at different date and sidereal time were obtained by solar spectrum calculator on the website of PV lighthouse. The module location was assumed to be in Shanghai, namely latitude of 31° and longitude of 121°. The modules were fixed at latitude tilt and faced due south. Other parameters were kept the default values.

## Conflict of Interest

The authors declare no conflict of interest.

## Supporting information

Supplementary
