# Supplementary material for: Realization of Quasi‐Omnidirectional Solar Cells with Superior Electrical Performance by All‐Solution‐Processed Si Nanopyramids
Source: Adv Sci (Weinh). 2017 Jul 6;4(11):1700200. doi: 10.1002/advs.201700200 (PMC5700634; doi:10.1002/advs.201700200)
Supplement: Supplementary file 1 — Supplementary [file ADVS-4-na-s001.pdf]

Copyright WILEY-VCH Verlag GmbH & Co. KGaA, 69469 Weinheim, Germany, 2017.

Supporting Information

for *Adv. Sci.,* DOI: 10.1002/advs.201700200

Realization of Quasi-Omnidirectional Solar Cells with Superior Electrical Performance by All-Solution-Processed Si Nanopyramids

*Sihua Zhong, Wenjie Wang, Miao Tan, Yufeng Zhuang, and Wenzhong Shen**

Supporting information

Realization of quasi-omnidirectional solar cells with superior electrical performance by all-solution-processed Si nanopyramids

Sihua Zhong[1], Wenjie Wang[1], Miao Tan[1], Yufeng Zhuang[1] and Wenzhong Shen[1,2,*]

[1] *Institute of Solar Energy, and Key Laboratory of Artificial Structures and Quantum Control (Ministry of Education), Department of Physics and Astronomy, Shanghai Jiao Tong University, Shanghai 200240, People's Republic of China*

[2] *Collaborative Innovation Center of Advanced Microstructures, Nanjing 210093, People's Republic of China*

* *Corresponding author, E-mail: wzshen@sjtu.edu.cn*

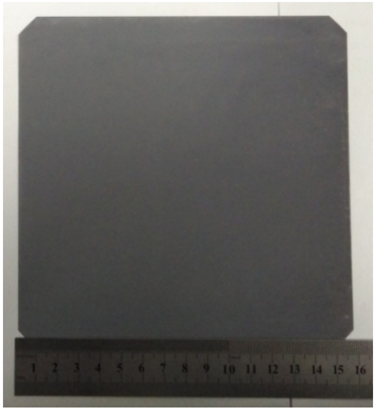

Figure S1 Digital photograph of a large-sized (156 mm × 156 mm) wafer with surface textured by MAAE method. Form the figure, it can be seen that the surface is uniform.

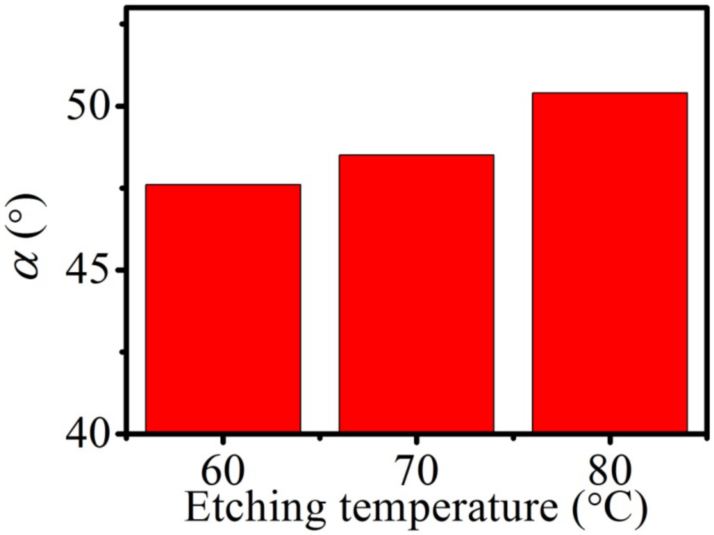

Figure S2 Base angles (αs) of Si pyramids fabricated by the MAAE method varying with etching temperature. It can be seen that higher temperature results in higher α, suggesting stronger anisotropic etching.

Table S1 Averaged cell performances of SiMPs-textured heterojunction solar cells (4 cells), SiNPs-textured heterojunction solar cells (5 cells), SiMPs-textured homojunction solar cells (10 cells) and SiNPs-textured homojunction solar cells (11 cells). V_{OC}, J_{SC}, FF, η and R_S represent open circuit voltage, short circuit current density, fill factor, conversion efficiency and series resistance, respectively.

Type of cells	V_{OC} (mV)	J_{SC} (mA/cm^2)	FF (%)	η (%)	R_S (mΩ)
SiMPs-heterojunction	721	37.1	76.4	20.4	4.2
SiNPs-heterojunction	729	36.0	75.3	19.8	5.4
SiMPs-homojunction	636	38.5	80.3	19.7	2.4
SiNPs-homojunction	640	37.8	80.9	19.6	2.4

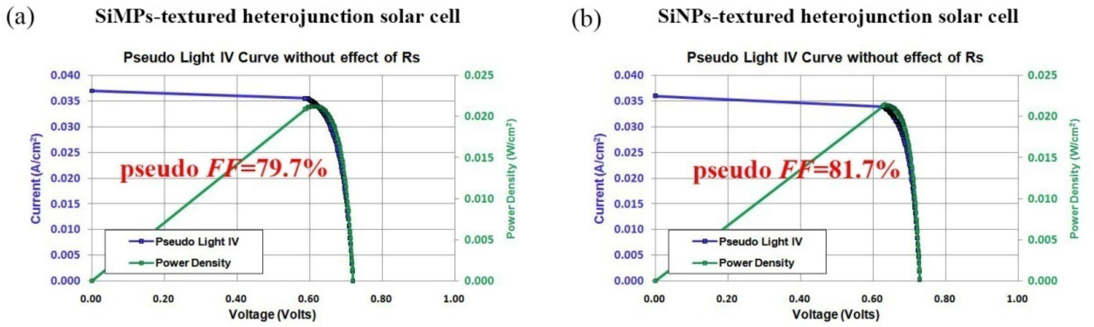

Figure S3 Pseudo current-voltage curves of (a) SiMPs-textured and (b) SiNPs-textured heterojunction solar cells measured by Suns-*V*oc. The result demonstrates that SiNPs-textured heterojunction solar cell has higher *FF* if without considering the effect of R_s.

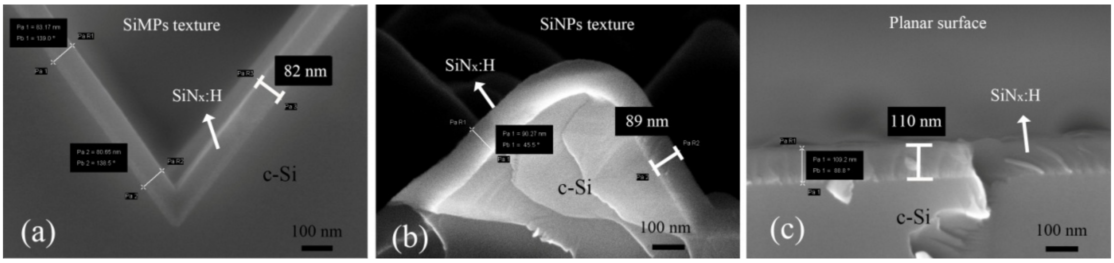

Figure S4 Comparison of thicknesses of SiN_x:H films on different surfaces deposited with the same condition by plasma enhanced chemical vapor deposition. (a) About 82 nm on SiMPs-textured surface. (b) About 89 nm on SiNPs-textured surface. (c) About 110 nm on planar surface. Obviously, the smaller the surface, the thicker the SiN_x:H layer (Surface area: SiMPs-textured surface > SiNPs-textured surface > planar surface).

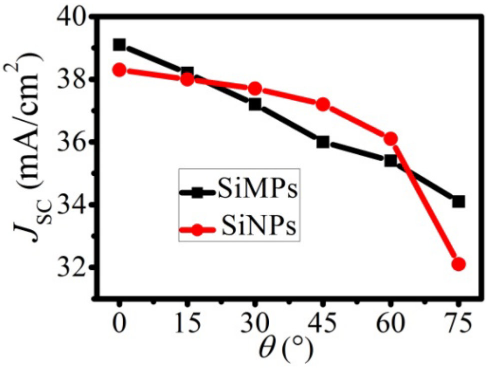

Figure S5 Comparison of the calculated J_{SC}s of the SiMPs-textured and SiNPs-textured heterojunction solar cells varying with θ. Note that the J_{SC}s are calculated by integrating QE curves over AM1.5 solar spectrum (300-1200 nm) and assuming that the incident photon amounts are the same for all θs. It can be seen that the J_{SC} of the SiMPs-textured solar cell decreases almost linearly with increasing θ. Nevertheless, the J_{SC} of the SiNPs-textured one decreases much slowly with increasing θ in the region of 0--45°, but outside the region, its decrease accelerates and becomes sharply when θ is larger than 60°. Obviously, the variations of J_{SC}s with θ are similar to those of QEs at 900 nm.

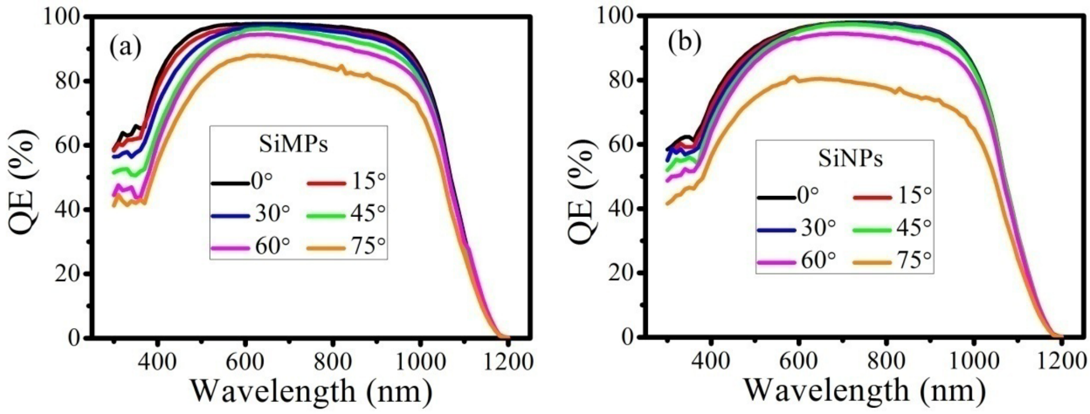

Figure S6 QE spectra of (a) SiMPs-textured (b) SiNPs-textured homojunction solar cell as a function of θ. Exhibiting the same behaviors with those of heterojunction solar cells, QE spectra of the SiMPs-textured homojunction solar cell decrease obviously with increasing θ. Nevertheless, QE spectra of the SiNPs-textured homojunction solar cell hardly decrease with increasing θ in the region of 0--45°, but become to drop sharply when θ is larger than 60°.
